# Are Health Facility Management Committees in Kenya ready to implement financial management tasks: findings from a nationally representative survey

**DOI:** 10.1186/1472-6963-13-404

**Published:** 2013-10-10

**Authors:** Evelyn Waweru, Antony Opwora, Mitsuru Toda, Greg Fegan, Tansy Edwards, Catherine Goodman, Sassy Molyneux

**Affiliations:** 1 Kenya Medical Research Institute - Wellcome Trust Research Programme, Nairobi, Kenya; 2 Centre for Tropical Medicine, Nuffield Department of Clinical Medicine, University of Oxford, CCVTM, Oxford OX3 7LJ, UK; 3 Department for Infectious and Tropical Diseases, London School of Hygiene & Tropical Medicine, London, UK; 4 Department for Global Health and Development, London School of Hygiene & Tropical Medicine, London, UK

**Keywords:** Health facility management committees, Direct facility funding, Community participation, Community accountability

## Abstract

**Background:**

Community participation in peripheral public health facilities has in many countries focused on including community representatives in Health Facility Management Committees (HFMCs). In Kenya, HFMC roles are being expanded with the phased implementation of the Health Sector Services Fund (HSSF). Under HSSF, HFMCs manage facility funds which are dispersed directly from central level into facility bank accounts. We assessed how prepared HFMCs were to undertake this new role in advance of HSSF roll out, and considered the implications for Kenya and other similar settings.

**Methods:**

Data were collected through a nationally representative sample of 248 public health centres and dispensaries in 24 districts in 2010. Data collection included surveys with in-charges (n = 248), HFMC members (n = 464) and facility users (n = 698), and record reviews. These data were supplemented by semi-structured interviews with district health managers in each district.

**Results:**

Some findings supported preparedness of HFMCs to take on their new roles. Most facilities had bank accounts and HFMCs which met regularly. HFMC members and in-charges generally reported positive relationships, and HFMC members expressed high levels of motivation and job satisfaction. Challenges included users’ low awareness of HFMCs, lack of training and clarity in roles among HFMCs, and some indications of strained relations with in-charges. Such challenges are likely to be common to many similar settings, and are therefore important considerations for any health facility based initiatives involving HFMCs.

**Conclusion:**

Most HFMCs have the basic requirements to operate. However to manage their own budgets effectively and meet their allocated roles in HSSF implementation, greater emphasis is needed on financial management training, targeted supportive supervision, and greater community awareness and participation. Once new budget management roles are fully established, qualitative and quantitative research on how HFMCs are adapting to their expanded roles, especially in financial management, would be valuable in informing similar financing mechanisms in Kenya and beyond.

## Background

### Role of health facility management committees in peripheral health facilities

There is a recognised need to improve the quality and utilisation of services provided by public primary care facilities in developing countries [1]. Health facility management committees (HFMCs) are considered one mechanism for leveraging such health system change, by encouraging direct engagement of communities in health facility activities [2-4]. HFMCs in many developing country settings were initially introduced several decades ago, as part of wider reorganisation of the health system based on principles of decentralisation, community participation and inter-sectoral collaboration [5,6]. The establishment of structures closer to service users, and inclusion of community representatives in those structures, was aimed at ensuring local problems were more easily seen or voiced, and responded to [7].

The potential for mechanisms such as HFMCs to meet their goals has been limited by wider decentralisation challenges such as insufficient transfer in practice of decision-making power to local levels for a range of functions, lack of clarity in responsibilities at local levels, and broader factors such as the prevailing political context, and inadequate access to financial resources [8-10]. Further challenges across many settings have included problems with the selection and functioning of committees, lack of clarity in roles and responsibilities, difficulty in sustaining voluntary membership over time, insufficient resources, inadequate representation of and links with the wider community, and inadequate interest in and support for involving communities among key health workers or managers [3,4,11]. There are also potential negative consequences with direct involvement of the public in health facility functioning, including real or perceived manipulation of communities or of health facilities and their funds by inappropriately selected or trained committee members, or by politicians and other locally prominent persons. Such challenges may in turn lead to inappropriate use of scarce health system funds, and deterioration in relations between the public and health systems.

### Kenyan context: health sector reforms and the Health Sector Services Fund

Kenya has been reforming its health sector for decades. A government policy introduced in the early 1980s identified the district as the most basic and effective unit for planning, development and delivery of public services; an approach that was supported through the 1990s and early 2000s through The Kenya Health Policy Framework of 1994, and the National Health Sector Strategic Plans of 1999–2004 and 2005–2010. These frameworks and plans included as a strategic imperative the creation of 'an enabling environment for increased private sector and community involvement in health sector provision and finance’ [12]; with the latter implemented in part through strengthening the capacity of HFMCs, which include the facility in-charge and community members elected from the facility catchment area [13].

Health system financing mechanisms in Kenya have also changed over time. Public health centres and dispensaries have always controlled relatively few resources: construction, qualified health staff, drugs and other equipment are all supplied from the centre in kind; while money which is supposed to come from the centre to cover other costs such as support staff, maintenance, allowances, fuel, and non-medical supplies has often failed to trickle down from central level to these facilities [14]. User fees were introduced as a source of additional health facility financing in the 1980s, with HFMCs overseeing expenditure of 75% of cash raised through user charges within facilities. In practice, facilities have often had to rely on these funds to cover costs of items that are supposed to be provided centrally [6]. As in other countries, user fees also had the negative effect of reducing access of health care for the poor [15]. In 2004 the '10/20’ policy was introduced with the aim of reducing user fees in dispensaries and health centres to 10 and 20 Kenyan shillings respectively, but many facilities have not strictly adhered to this policy [4,16].

An innovative finance initiative with the potential to strengthen community accountability and improve financing of the lower levels of the health system is the Health Sector Services Fund (HSSF). Under HSSF, the Government and development partners contribute to a central fund, which is used to credit funds directly into approved facilities’ bank accounts. At the facility level, HSSF funds are managed by an HFMC that includes community members from the facility catchment area. HSSF therefore provides HFMCs with budgets to manage.

HSSF was piloted in Coast Province starting in 2005 [3,6,17] and in 2010 national roll out began in phases. In October 2010, funds were credited to all 590 public health centres, with further roll out to dispensaries taking place in 2012. HSSF funds are intended to cover the facility’s operational expenses according to financial guidelines set out by the Ministry for Public Health and Sanitation (MOPHS) [18]. The Ministry continues to provide facility infrastructure, trained health workers, drug kits, and medical supplies directly to facilities.

### Expanding responsibilities for health facility management committees in Kenya

Different forms of HFMCs have been in place in Kenya since the 1980s [5,6]. The National Health Sector Strategic Plans [19] required all public health centres and dispensaries to establish committees, with the roles, responsibilities, and powers outlined in Table 1. A key role of facility committees was to oversee general operations and management of facilities. With user fees being an important revenue source for facilities in Kenya, this included overseeing the use of user fee revenues collected at the facility in order to increase community accountability in the way that facility funds were used.

**Table 1:** Extracts from key government regulations concerning operation of health facility management committees

**Document**	**Topic addressed**	**Extract**
Best practices in community-based health initiatives [21].	Roles, responsibilities and powers– 1998	Roles and responsibilities
		1) To oversee the general operations and management of the health facility;
		2) To advise the community on matters related to the promotion of health services;
		3) To represent and articulate community interests on matters pertaining to health in local development forums;
		4) To facilitate a feedback process to the community pertaining to the operations and management of the health facility;
		5) To implement community decisions pertaining to their own health and;
		6) To mobilize community resources towards the development of health services within the area.
		Powers
		1) The committee shall have the authority to raise funds from within itself, the community or from donors and other well-wishers for the purpose of financing the operations and maintenance of the facility;
		2) The committee shall have authority to hire and fire subordinate staff employed by itself in the health facility;
		3) The committee shall oversee the development and expansion and maintenance of the physical facilities within their respective area.
Guidelines on the financial management for HSSF, Nov 2010 [20].	Financial roles of HFMCs	1) Supervise and control the administration of the funds allocated to the facilities;
		2) Open and operate a bank account at a bank approved by the Minister for the time being responsible for finance;
		3) Prepare work plans based on estimated expenditures;
		4) Cause to be kept basic books of accounts and records of accounts of the income, expenditure, assets and liabilities of the facility as prescribed by the officer administering the Fund;
		5) Prepare and submit certified periodic financial and performance reports as prescribed;
		6) Cause to be kept a permanent record of all its deliberations.
Guidelines on the financial management for HSSF, Nov 2010 [20].	Membership and selection of health facility management committees	a) A representative from the provincial administration in the area of jurisdiction;
		b) The person in charge of the health facility who shall be the secretary;
		c) The District Medical Officer of Health or his representative duly nominated by him in writing;
		d) The person in charge of the local authority health facilities or the area councillor;
		e) The following persons, who shall be residents of the area of jurisdiction, appointed by the Minister or any other person authorized by him in writing
		(i) One person who shall have knowledge and experience in finance and administration; and
		(ii) Four persons of whom three shall be women
		A person shall not be appointed as a member of a committee under item (e) unless that person holds at least form four level certificate of education or its equivalent.

To strengthen HFMC financial oversight roles, and preparedness for national implementation of HSSF, the financial aspects of committee roles and functions were clarified in 2007 and 2009 (Table 1), emphasising HFMC responsibilities for planning, managing and accounting for facility expenditure [13,18,20]. The required composition of HFMCs was also modified in 2009 by Government Gazette (an official notice required for all new legislation), reducing the number of community committee members from ten to five. It instructed that the five community members selected should include: one person who has knowledge and experience in finance and administration and three women (Table 1, row e). Representatives from the provincial administration and local authority were also added to the total number of committee members (Table 1, row a) [13,21].

Findings from an evaluation of the Coast pilot of HSSF suggested that HFMCs were generally functioning well and played an important role in facility operations. The breadth and depth of engagement had also reportedly increased after the introduction of direct funding of health facilities. Although HFMCs had previously been involved in management of user fee revenues, the total amount of funds they were managing increased with HSSF. To optimise their contribution, it was argued that efforts were needed to improve HFMC training, clarify their roles, and strengthen engagement with the wider community [3,17].

Given the important role of HFMCs in HSSF, it is essential for national implementation that the committees are in place, and that they have the training and role awareness that is key to their ability to manage budgets effectively. In this paper, we use a large scale quantitative facility based survey to describe HFMC’s readiness for their financial management tasks in advance of national HSSF roll out, and to consider the relevance for other similar settings. Drawing on McCoy et al’s review [11] and our own previous work [3,4] we identified a set of key factors that could be quantitatively measured at the facility level that would affect the potential of HFMCs to effectively perform their new financial management roles (Table 1). These factors comprised committee composition (selection, tenure, and constitution); operations of HFMCs (back accounts, training and meetings); HFMC links to the broader communities that they are expected to represent; awareness of HFMC roles among members, health workers managing facilities (in charges), and users; and HFMC members’ motivation, job satisfaction, and relationships with in-charges. In describing and discussing our data on these issues, we contribute to the relatively small body of empirical data on mechanisms to strengthen community involvement in peripheral health facilities in low and middle income countries [3,4,11].

## Methods

The data presented in this paper were collected through a nationally representative baseline survey of public sector health centres and dispensaries commissioned by the Ministry of Public Health and Sanitation (MOPHS). The baseline survey is part of a wider evaluation of HSSF in Kenya, for which the overall report and data collection tools are available elsewhere (http://resyst.lshtm.ac.uk/resources/nationally-representative-survey-kenya's-public-sectorhealth-centres-and-dispensaries) [17].

We conducted the baseline survey in facilities and their HFMCs across all 8 Kenyan provinces following a 2-stage sampling process. First, we randomly selected 3 districts per province in 7 provinces (excluding Nairobi; n = 21 districts), and one district from each of the 3 municipal areas (Nairobi, Mombasa and Kisumu). Within each selected district, our sampling frame included all government-owned health centres and dispensaries eligible to receive HSSF, which was almost all public facilities (for detailed criteria, see [20]); facilities also had to be operational, and have at least one qualified health worker at the time of the survey. Next, we stratified the sample by facility type (health centre and dispensary), and for each facility type, randomly selected 7 facilities per district. In districts with less than 8 facilities of a given type, we surveyed all relevant facilities in that district.

Data were collected between July and September 2010, before HSSF funds began to flow to peripheral facilities. A structured survey was conducted at each selected facility which included an interview with the facility in-charge, and a self-administered questionnaire for the in-charge on motivation and empowerment. Interviews were conducted with two 'ordinary’ community members of the selected HFMCs; that is, the category of interviewees in row e) of Table 1. These HFMC members were identified and invited for interview by the in-charge, who was requested to include both an office and non-office holder if possible. Exit interviews were conducted with a convenience sample of 3 outpatients or their caretakers who had come to the facility for curative care [17]. In total we held interviews with 248 in-charges, 464 HFMC members and 698 facility users (Table 2).

**Table 2:** Summary of data collected

	**Non-municipal**		**Municipal**		**Total**
	**Dispensaries**	**Health centres**	**Dispensaries**	**Health centres**	
In-charge questionnaire	144	65	21	18	248
In-charge SAQ	141	65	21	18	245^a^
HFMC member questionnaire	279	126	32	27	464^b^
Exit interview questionnaire	400	192	53	53	698^c^
District context tool	21		3		24

HFMC = health facility management committee.

^a^ SAQ = self-administered questionnaire, 3 SAQs were not returned to interviewers.

^b^ HFMC members: The intended number at 2 per facility was 496. Both HFMC members were interviewed in 226 facilities (n = 452 HFMC members); only one HFMC member was interviewed in 12 facilities (n = 12), and no HFMC members were interviewed in 10 facilities (2: no HFMC, 8: members unavailable for interviews).

^c^ Exit Interviews: the intended number at 3 per facility was 744 patient records. 753 patients were approached; 50 declined to be interviewed, 3 did not meet the inclusion criteria, and 2 were later excluded because they were unable to answer the questions consistently. In total data were collected from 698 patients.

Across the data collection tools [17], we collected information where relevant on HFMC selection, composition and functioning, HFMC members’ awareness of their roles, their perceptions of the benefits of being HFMC members, and their motivation and job satisfaction. These dimensions of HFMC functioning were chosen based on guidelines (Table 1), findings of the Coast pilot of direct facility funding [6], and characteristics of HFMCs that could be measured quantitatively in a nationally representative survey. Questions were structured using three main approaches, with specific questions and options refined through careful piloting in two districts^a^.

In addition to the above quantitative work, qualitative data were collected from MOPHS managers at the district level during individual or group interviews, as preferred by District Health Management Team (DHMT) members (Table 2). District health managers interviewed included the District Medical Officer of Health (DMOH), District Health Administrator, District Health Public Nurse, District Public Health Officer, and District Health Information and Records Officer. These interviews were designed to give a district perspective to the baseline study, and to collect contextual information. Data collected relevant for this paper were district managers’ views on HFMC selection and operations, and how these have changed over time, particularly since the government’s change of membership criteria.

Quantitative data were entered directly into mini-laptops in MS Access and later merged in MS Excel and imported into Stata version 11 for cleaning and data analysis [22]. Given the study design and sampling strategy, we accounted for variation in sampling probability across facilities using weights inversely proportional to population size*,* stratification by province and health facility type, and clustering at district and facility levels. Using this approach, we estimated prevalence on the percentage scale, with corresponding 95% CIs, for our key areas of interest as outlined above. Qualitative data from audio recordings were transcribed and analysed using the framework approach [23].

The study was approved by science and ethics committees of the Kenya Medical Research Institute and the London School of Hygiene and Tropical Medicine. Informed consent was obtained for all interviews.

## Results

Following an overview of the characteristics of the interviewees, we present information on HFMC characteristics, their operations, user awareness of their presence, perceptions of HFMC roles, and views of their motivation, job satisfaction and relations with health workers. Any differences observed between health centres and dispensaries, and between municipal and non-municipal districts are highlighted.

### Interviewee characteristics

#### In-charges

Three quarters (73.1%) of the 248 in-charges were aged between 25 and 44 years, and a quarter (26.5%) were aged 45 years or above, with only 0.4% aged less than 25. About half (47.9%) of the in-charges were female, although this proportion was higher in municipal areas where 75.3% and 66.7% were female in dispensaries and health centres, respectively. Most in-charges were qualified health workers, typically enrolled nurses (diploma level training; 47.8%) and registered nurses (bachelor’s degree in nursing; 29.5%), with 13% being clinical officers. A small proportion of the in-charges were community health workers (CHWs) (5.3%) or had other health qualifications (4.5%), such as retired nurse, laboratory technologist/technician, or public health technician.

#### HFMC members

All 464 HFMC members interviewed from the 248 facilities were aged 25 years or over, with just over half aged 45 years or above, and 23.3% female. Just over half (53.2%) had completed secondary school education, 29.4% had completed primary and 17.4% had not completed primary. The HFMC members interviewed were equally divided between chairpersons, treasurers and those not holding an office.

#### Exit interviewees

Among the 698 exit interviewees from the 248 facilities, 56.7% were seeking curative health services for themselves, and 43.3% for their sick children. Just over half were aged 25–44 years (54.5%), 27.8% were between the ages of 16–24 years and 17.7% were aged 45 years or above. Only 37.0% of all interviewees had completed primary school, but almost half (49.6%) reported literacy in English and 70.8% in Kiswahili. Almost two thirds (64.9%) were female, although this figure was lower in municipal dispensaries (47.4%).

### Committee composition

Fundamental to preparedness for HSSF is having a duly formed HFMC with the basic characteristics required by the government. Almost all facilities (97.2%) had HFMCs, with a median of 10 members per committee (Table 3). Three out of five (61.4%) facilities had all types of members specified in the Gazette. The in-charge, chief/assistant chief and ordinary community committee members were the most commonly cited committee members, with almost all HFMCs including ordinary community members. 30% of all committee members – but 40% of ordinary community committee members - were female.

**Table 3:** Characteristics and operations of health facility management committees

	**Non-municipal**		**Municipal**		**Total**
	**Dispensary**	**Health centre**	**Dispensary**	**Health centre**	
**N**	**144**	**65**	**21**	**18**	**248**
	**%**	**%**	**%**	**%**	**%**
	**(95% CI)**	**(95% CI)**	**(95% CI)**	**(95% CI)**	**(95% CI)**
Facility has HFMC	97.8	97.9	80.2	94.4	97.2
	(92.3 - 99.4)	(91.2 - 99.5)	(10.7 - 99.3)	(40.4 - 99.8)	(93.0 - 98.9)
**Of those with HFMCs**					
**N**	**140**	**63**	**18**	**17**	**238**
	**Median**	**Median**	**Median**	**Median**	**Median**
	**[IQR]**	**[IQR]**	**[IQR]**	**[IQR]**	**[IQR]**
**Median** number of committee members	10	9	8	9	10
	[9-11]	[8-11]	[7–9.5]	[9-11]	[9-11]
**Median** tenure (years)	3	3	3	3	3
	[3]	[3]	[3]	[3]	[3]
	**%**	**%**	**%**	**%**	**%**
	**(95% CI)**	**(95% CI)**	**(95% CI)**	**(95% CI)**	**(95% CI)**
HFMC included all types of members specified in Gazette (see Table 1)	58.0	81.1	51.4	58.8	61.4
	(39.2 - 74.8)	(65.7 - 90.5)	(8.9 - 92.0)	(7.8 - 96.0)	(44.7 - 75.8)
Percentage of all committee members that are female	30.8	28.6	33.3	33.3	30.0
	(23.1 - 40.0)	(23.1 - 33.3)	(22.5 - 50.0)	(28.6 - 50.0)	(23.1 - 40.0)
Percentage of ordinary community committee members that are female	40.0	40.0	40.0	50.0	40.0
	(30.0 - 50.0)	(33.3 - 50.0)	(37.5 - 50.0)	(30.0 - 60.0)	(31.3 - 50.0)
HFMCs with written constitution	55.4	38.5	60.0	76.5	53.5
	(34.4 - 55.3)	(27.3 - 51.0)	(9.7 - 95.4)	(27.0 - 96.6)	(44.0 - 62.8)
HFMCs with fixed tenure	92.7	92.2	71.2	80.0	92.0
	(82.4 - 97.2)	(82.4 - 96.8)	(10.1 - 98.2)	(5.4 - 99.6)	(83.6 - 96.3)
One or more ordinary community committee members trained in facility or financial management	15.5	26.7	20.2	29.4	17.5
	(6.9 - 31.3)	(7.8 - 61.0)	(3.4 - 64.9)	(3.7 - 81.8)	(8.7 - 32.1)
Held full committee meeting in the last quarter	76.6	86.3	70.2	76.5	77.9
	(58.8 - 8.2)	(75.0 - 3.0)	(20.5 - 95.6)	(15.2 - 98.3)	(62.9 - 88.0)
Held executive meeting in the last quarter	48.0	61.6	44.2	29.4	49.8
	(39.5 - 56.6)	(42.7 - 77.6)	(9.8 - 85.2)	(5.1 - 76.3	(41.4 - 58.1)

CI = confidence interval; HFMC = health facility management committee; and IQR = inter-quartile range.

Source: In charge interviews.

Regarding selection of ordinary community committee members, most were reported by in-charges to have been selected at a 'baraza’ (a Kiswahili word for public meeting) (69.0%), or to have been nominated by the District Medical Officer of Health (DMOH) (9.2%). Very few were nominated by community leaders (3.9%), in-charges (1.6%) or village health committees (0.6%). In 6.1% of facilities with HFMCs, in-charges did not know how ordinary community members of HFMCs were selected (ranging from 3.9% of non-municipal dispensaries to 16.9% of non-municipal health centres). Qualitative interviews with district managers indicated that following the recent Gazette notice [18] there had been a shift with fewer HFMC members being selected by baraza, and more being nominated by authority figures such as the in-charge, the DMOH or the chief. When asked how this had affected HFMC performance, managers were equally divided between those who felt it had made no difference, and those who thought that the new HFMCs were working better than the previous ones. Only managers in one out of the 24 districts selected reported that the previous HFMCs had functioned better.

HFMC operations should be guided by a written constitution which outlines HFMC members’ roles, requirements for training, frequency of meetings and tenure. Of facilities with HFMCs (97.2%), only 53.5% had a written constitution for the committee (Table 3). Most HFMCs (92.0%) had a fixed tenure of 3 years as stipulated by the Gazette [18].

### Operations of HFMCs: bank accounts, training, and meetings

In-charge interviews indicated that bank accounts were held by all non-municipal health centres, 87.7% of non-municipal dispensaries, and 72.2% of municipal health centres, but only 54.7% of municipal dispensaries. The proportion of facilities with bank accounts in Nairobi was particularly low (14.8%), while the proportions in all other provinces exceeded 80%.

In only 17.5% of facilities with HFMCs was one or more ordinary community committee member reported to have received training in facility or financial management (Table 3). However 77.9% of facilities with HFMCs had held full committee meetings in the last quarter (median = 1 meeting, IQR 1–2), and half had held smaller executive meetings of the HFMC office holders (median = 1 meeting). Overall, there were more full committee meetings held in the last quarter among municipal health centres (median = 2) and dispensaries (median = 2), than among non-municipal health centres (median = 1) and dispensaries (median = 1).

### HFMC links to the broader communities they represent

Almost all facilities (97.2%) had some facility information displayed and visible for users, such as data on services offered (37.1%), official user fees (25.7%), and facility utilization (9.4%). However, names of HFMC members were displayed in only 4.4% of facilities (none of which were municipal) facility expenditure in only 1.5%, and facility income in only 1.3%.

Almost half (44.5%) of all users interviewed were aware of the existence of a HFMC (Table 4). This awareness was generally highest among users of non-municipal facilities. Of those aware of the existence of HFMCs, 77.8% were aware that ordinary community members sit on the committee, just over half (54.2%) knew the chair and almost three quarters (74.8%) knew a member (but not necessarily by name).

**Table 4:** User awareness of Health Facility Management Committees

	**Non-municipal**		**Municipal**		**Total**
	**Dispensary**	**Health centre**	**Dispensary**	**Health centre**	
**N**	**398**	**192**	**53**	**53**	**696**
	**%**	**%**	**%**	**%**	**%**
	**(95% CI)**	**(95% CI)**	**(95% CI)**	**(95% CI)**	**(95% CI)**
Aware of existence of HFMCs	47.4	38.1	16.5	12.8	44.5
	(40.9 – 53.9)	(30.4 – 46.5)	(2.9 – 57.1)	(3.0 – 41.3)	(39.3 – 49.8)
**Of those aware of the existence of HFMCs:**					
**N**	**166**	**73**	**9**	**7**	**255**
Know HFMC Chair	56.0	44.5	42.0	40.9	54.2
	(49.1 – 62.8)	(34.2 – 55.3)	(2.3 – 95.6)	(4.1 – 91.8)	(48.2 – 60.1)
Know any HFMC member	77.0	65.2	42.0	40.9	74.8
	(70.2 – 82.6)	(56.2 – 73.1)	(7.2 – 87.1)	(4.1 – 91.8)	(68.6 – 80.1)
Aware that ordinary community members are on HFMC ^a^	79.1	71.9	65.3	40.9	77.8
	(72.1 – 84.7)	(54.9 – 84.3)	(7.7 – 97.7)	(2.9 – 94.1)	(70.6 – 83.6)

CI = confidence interval; and HFMC = health facility management committee.

^a^ Data were missing for 5 exit interviewees.

Source: Patient exit interviews.

### Awareness of HFMC roles: HFMC members, in-charges and users

Given the importance of clarity in roles for HFMC functioning [3,4], we asked in-charges, HFMC members and users to go through a checklist of potential HFMC roles and indicate which ones they thought were correct. In developing this checklist, we considered key roles which would enable HFMCs to achieve their general and financial responsibilities (Table 1). These included raising issues they have heard in the community with facility staff, employing new support staff such as cleaners and watchmen for the facility, and determining how facility funds are utilized. We also included important potential roles for which there appeared to be lack of clarity in official roles and responsibilities (whether they are able to set the level of user fee charges; and whether they should supervise health facility staff) [3,4,16,21], and one role which currently remains firmly in the hands of MOPHS management (employment of new government health workers such as nurses).

**Figure 1:**
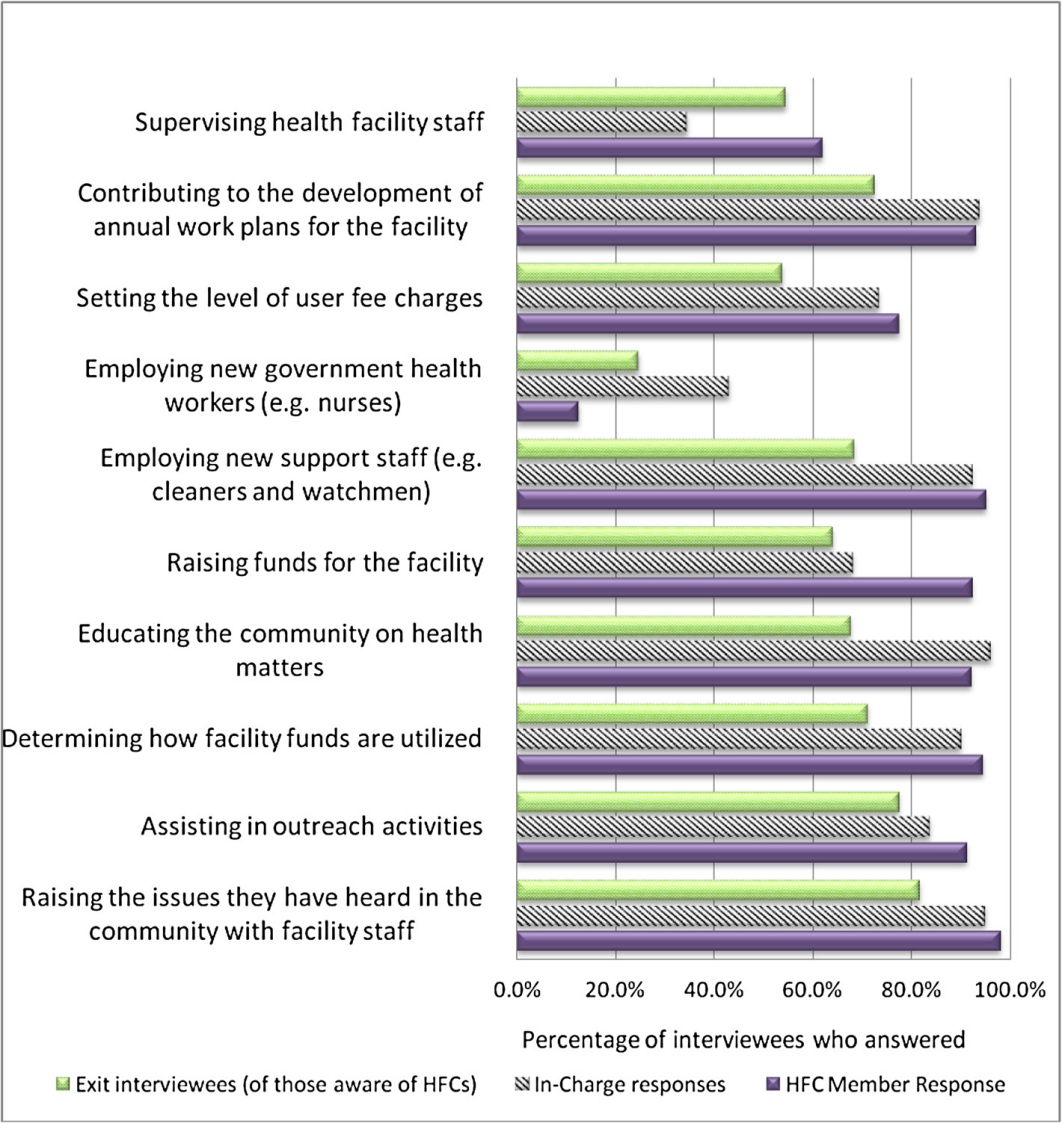
Perception of HFMC roles by exit interviewees, In-charges and HFMC members (% who answered yes to the question: Do you think that the following are the roles of the HFMC?).

The perceptions of in-charges, HFMC members and users on these roles are shown in Figure 1. Virtually all HFMC members perceived their roles as including: raising issues they heard in the community with facility staff (98.0%); employing new support staff such as cleaners and watchmen (95.1%); determining how facility funds are utilized (94.5%); contributing to the development of annual work plans for the facility (93.1%); raising funds for the facility (92.3%); assisting in outreach activities (91.2%); and educating the community on health matters (92.1%). The majority also mentioned setting the level of user fee charges (77.5%), and supervising health facility staff (61.9%) as roles of the HFMC. Only 12.5% of HFMC members perceived employing new government health workers such as nurses as a role of the HFMC.

In-charges with a HFMC described HFMC roles in similar ways, but a much lower percentage considered supervision of facility staff a HFMC role (34.5%). Users of facilities often did not know whether stated roles were indeed HFMC responsibilities. For example, of the 44.5% (n = 696) who were aware that an HFMC existed, a significant proportion reported that they did not know whether it was the HFMC’s role to: set the level of user fees (24.7%), contribute to the development of annual work plans (22.3%) or decide on how facility funds are utilized (19.3%).

### HFMC motivation, job satisfaction, and relations with the in-charge

Key factors influencing HFMC functioning in other low and middle-income countries - beyond basic HFMC characteristics, training, and clarity in roles and responsibilities - are HFMC members’ motivation and job satisfaction [3,4,6,11]. To explore this, HFMC members were asked about the benefits of being HFMC members, and presented with a series of statements to which they were asked to respond on a five-point Likert scale (strongly agree, agree, neutral, disagree or strongly disagree). The statements were based on 6 domains: self-efficacy (confidence in their ability to perform their role), availability of organizational resources, financial rewards, relationship with the in-charge, relationship with the community, and overall motivation/job satisfaction [17]. Statements to document these domains, including negative questions, were randomly distributed within this section of the questionnaire. Similar questions on the relationship between community members of the HFMC and the facility in–charge were also put to in-charges.

**Figure 2:**
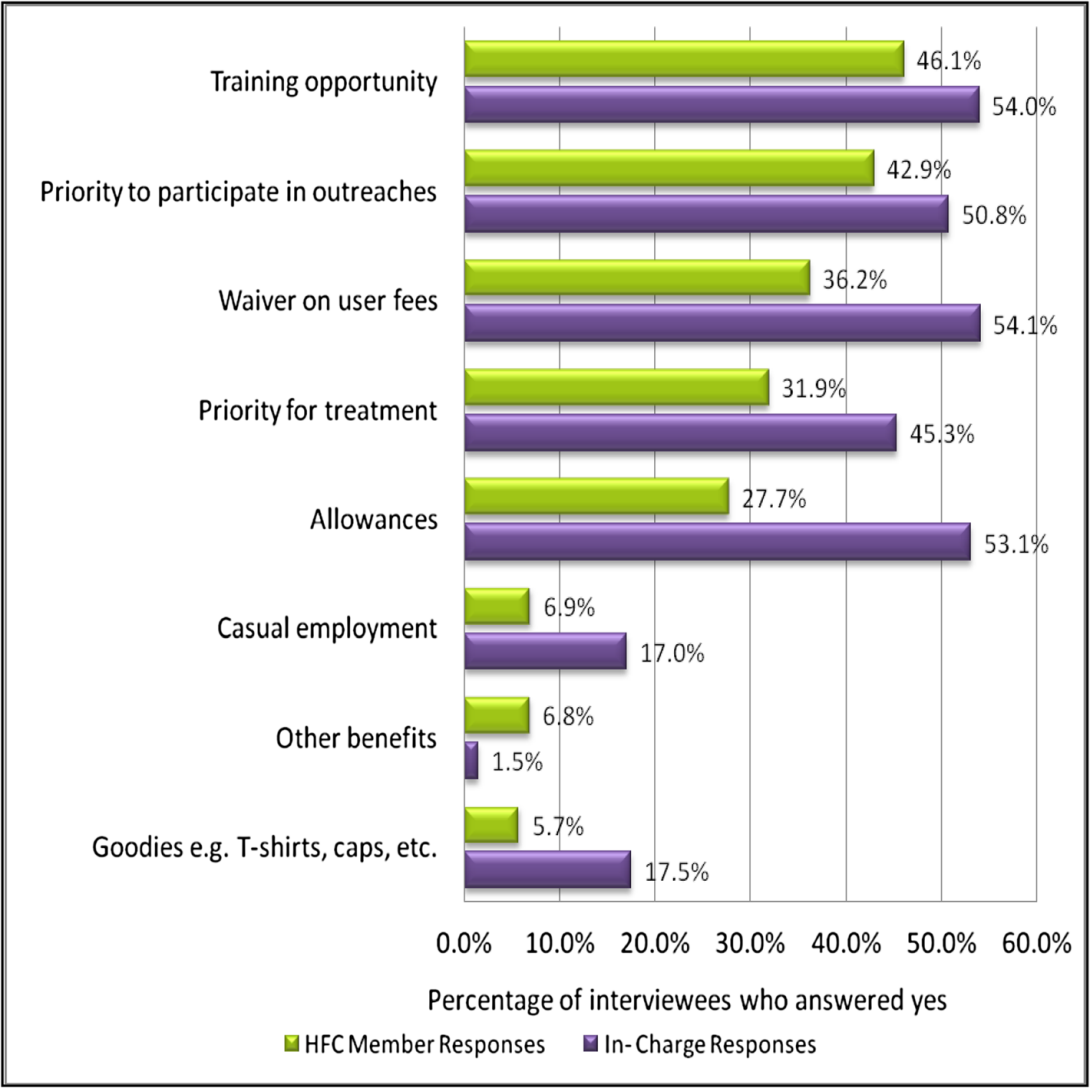
Benefits of being a HFMC member reported by HFMC members and In-charges (% who provided specific responses to the question: Have you received any benefits for being a committee member?).

The benefits of being a HFMC member reported by facility in-charges and HFMC members are shown in Figure 2. HFMC members’ most commonly cited benefits were training (46.1%) and opportunities to participate in outreaches and campaigns (42.9%). Around a third of HFMC members also mentioned waivers on user fees (36.2%), priority for treatment (31.9%), and allowances (27.7%). Each of these benefits was also mentioned by around a half of the in-charges.

Allowances were not mentioned as a benefit by HFMC members, although approximately half (53.1%) of the in-charges from facilities with HFMCs reported that allowances were given for full committee meetings, with allowances more common in non-municipal areas and in health centres (79.7% in non-municipal and 41.2% in municipal health centres; compared to 49.5% in non-municipal and 14.4% in municipal dispensaries). A little under a third of facilities with HFMCs (29.5%) gave allowances for executive committee meetings, with the proportion substantially higher in non-municipal facilities (28.5% of dispensaries and 40.8% of health centres) than municipal facilities (2.4% of dispensaries and 5.9% of health centres). Where allowances were given, the median figure was 200 Kenyan shillings (2.2 USD, 2010) per meeting for both full committee and executive committee meetings.

In general, most HFMC members appeared to be highly motivated, answering that they strongly agreed or agreed with the statements: “I am proud to be HFMC member” (99.1%); “being a HFMC member makes me feel good about myself” (96.9%); and “I am satisfied that I accomplish something worthwhile in this job” (97.7%). However, among HFMC members in municipal health centres, 7.4% strongly disagreed with the statement “I am satisfied that I accomplish something worthwhile in this job”. Noteworthy is that two thirds of HFMC members strongly disagreed with the statement that they get sufficient allowances for HFMC meetings (66.3%) and for outreach services (65.6%).

A generally positive relationship between the community members of the HFMC and the facility in–charges was indicated by HFMC members’ very strong support of the following statements (strongly agree or agree):

• It is useful to hear the views of the facility in–charge during HFMC meetings (99.7%);

• I believe that the in–charge works in the interest of this facility (93.0%);

• The health workers and the community members of the HFMC work well together (92.0%);

• If we have better knowledge, the facility in–charge is willing to accept advice from community members in the HFMC (93.5%).

Similarly, when facility in-charges were asked to describe the relationship between community members of the HFMC and the facility in–charge, most in-charges felt positive about relationships with the HFMC, with over 80% agreeing or strongly agreeing with statements that: “Committee members from the community made an important contribution to the last facility annual work plan”; “it is useful to hear the views of the HFMC members”; and “I trust the HFMC to work in the interest of this facility”.

Nevertheless, there were some HFMC members and in-charges that either agreed or strongly agreed with the following statements:

• “The facility in–charge sometimes looks down on community members in the HFMC” (11.5% HFMCs); and

• “Tensions between the in–charge and committee members undermine the achievements of the committee” (13.9% HFMCs; 47.9% of in-charges).

## Discussion

We conducted a nationally representative survey to assess preparedness of HFMCs for their new financial management roles under HSSF, and to consider the relevance of the findings for other similar settings and initiatives.

Kenya appears to have good potential with regards to HFMCs managing facility budgets, relative to other settings. For example, committees are in place and meeting regularly; there is general agreement among key players on their roles; HFMC members generally report being motivated and satisfied; and relations between facility in-charges and committee members appear relatively strong. However there were some differences between municipal and non-municipal facilities, including fewer municipal facilities with HFMC controlled bank accounts at the time of interview, and fewer municipal dispensaries – but not health centres - with HFMCs. The reasons for these differences need further exploration but might include facilities being controlled by the Nairobi Municipal Council rather than the Ministry of Public Health and Sanitation, and some facilities being owned by parastatal institutions such as the airport or prisons. There may also be difficulties in selecting HFMC members from highly diverse and mobile urban populations. This finding may change over time, but suggests that in Kenya and elsewhere, there should be careful consideration in HFMC initiatives of differences between rural and urban areas in socio-economic, cultural, and institutional contexts.

Across rural and urban settings, we identified some important challenges for HFMCs, which indicated problems in two key areas: selection and representation; and functioning. Selection of appropriate individuals to sit on HFMCs can be very complex, given that communities within catchment areas of facilities are far from homogenous (differing for example in age, gender, ethnicity and socio-economic status), and mechanisms for selecting individuals to represent those communities can be highly problematic [4]. Once selected, representatives should ideally then continue to interact with the communities they represent [4]. In our survey, nearly half of the HFMC members interviewed (46.8%) had not completed secondary education, suggesting that guidelines on educational status (Table 1) had not been followed. However, the gross secondary school enrolment rate in Kenya is low (22.5% in 2000; ranging from only 4.5% in North Eastern Province to 37.7% in Central Province), indicating that strict adherence to the guidelines could be practically difficult in certain locations and potentially undermine committee members’ representativeness of catchment populations in terms of their socio-economic, educational and demographic characteristics [24]. This suggests the requirement for all community members on HFMCs to have secondary school education should be reconsidered, particularly for some parts of the country. More broadly, this issue suggests the need in Kenya and elsewhere to carefully consider the ways in which committee members are expected to represent the communities they come from. Selection processes in many settings suggest that members are generally nominated or elected to interact with health managers *on behalf of* the communities they are selected from, as opposed to through being *typical members* of those communities. While this may be advantageous in technical aspects of the work such as budget oversight, it risks under-representation of the most vulnerable groups such as those without an opportunity for formal education, often the key target groups for public health services. Careful consideration of the intended balance between these different forms of representation, and ensuring the selection process supports this balance, would potentially assist.

One possible approach to minimize the challenge of inadequate representation of the most vulnerable groups on committees is to ensure that those populations are aware of the committee and are able to approach committee members. The majority of HFMC members in our survey reported being selected in a public meeting. However, relatively few users in our survey were aware of HFMCs and their roles, indicating a disconnect between committees and the broader community that has been observed elsewhere [3,4,25]. Although in our setting this problem might be attributable to the recent change in HFMC composition (Table 1), a similar challenge was noted in the evaluation of the HSSF pilot in Coast Province before these new committees had been introduced [3]. Moreover, only a fifth of the HFMC members we interviewed were recruited within the year before the interview. The problem is therefore more likely to be related to inadequate public awareness of the selection process, the selection process having taken place too long ago, and/or inadequate mechanisms for continued communication between HFMCs and the wider community. Further investigation of the relationship between the HFMC and members of the wider community is warranted. However findings in this paper for Kenya and elsewhere suggest the importance of initiatives aimed at strengthening community awareness of HFMCs and their roles, including through the provision of basic information such as names and contacts of HFMC members displayed in a user friendly manner on facility walls [3].

With regards to functioning of HFMCs, common challenges noted in other settings include members’ lack of clarity in roles and responsibilities, avoiding politicization, dilemmas related to voluntary participation/remuneration, information and resource asymmetries between health staff and community representatives, and building trustful relationships in these contexts [3,4]. The observation in our survey of some lack of clarity in roles among the HFMCs themselves, inadequate training of HFMCs, and some indications of strained relations with in-charges, all have negative implications for HFMC functioning, and therefore for HFMCs’ ability to take on broader roles such as budget management. These challenges were also noted in the previous study on HFMCs *after* the Coast pilot of HSSF [3,6], suggesting that these problems can persist, and may even be exacerbated, after additional funds that can be used relatively flexibly – as is the case with HSSF - are introduced.

While most HFMC members reported high levels of motivation, allowances were reported as a benefit less often than expected. This may have been due to a perception of allowances as more of a compensation for time or payment for service than a benefit. Moreover, the Swahili translation of “allowances” in the questionnaire (“*marupurupu ya pesa*”) may have been interpreted as extra amounts of cash in addition to the regular HFMC allowances. HFMC members may also have wished to de-emphasise any financial motivation they had for being committee members. In fact, the majority expressed dissatisfaction with the level of allowances. Indeed, there were no allowances for attending HFMC meetings in almost half of facilities. These findings suggest that as HFMC members gain additional responsibilities under HSSF it will be important to ensure that they feel adequately rewarded for their input, and this may require both financial and non-financial incentives. With regards to allowances, some consideration is needed as to whether these should be consistent across committees, and whether amounts paid are sufficient for sustained participation. For Kenya and elsewhere, the challenge is similar to that discussed more widely in the CHW literature [26,27], of ensuring that there is an appropriate balance between extrinsic motives – both financial and non-financial – and intrinsic motivation such as social recognition, knowledge gain, and the opportunity to make a social contribution. There is recognition that the former can crowd out the latter [27,28], with negative implications for members’ ability to adequately represent the most vulnerable groups who are most important to reach and hear, and for HFMC members’ ability to perform their other assigned roles.

The above findings suggest that there is some level of preparedness among HFMCs for broader financial management roles under HSSF, but also that there are some challenges which might limit the potential for the initiative to achieve its main instrumental goals: improved quality and utilisation [17]. Beyond the key aspects that we could measure quantitatively at the facility level, other influences on HFMC potential for financial management include the design and implementation of HSSF itself (such as content and quality of training, and the level and availability of resources at all levels); and the wider facility and community context, including for example the skills and attitudes of facility staff, district level support, internal accountability requirements up the hierarchy within the Ministries of Health and to other facility funders, and local political dynamics [11]. These factors in turn are influenced by wider changes in government operations within and beyond the health sector. In Kenya, the acceleration of the devolution of government functions from national level to 'semi-autonomous’ counties over the next few years will have major implications for all sectors including health in ways that are still being discussed. It is unclear how a vertical financing system like HSSF will be incorporated into the new county level.

Future quantitative and qualitative research should include efforts to document how HFMCs are coping with their new demands and if and how the HFMC limitations described above have been overcome. Specifically, it would be important to explore the impact of HSSF on HFMC operations and HFMC relations with community members and in-charges. Also valuable would be data on if and how HFMCs manage their planning and financial management responsibilities, and how any new developments, such as performance based financing, impact on this. Future mixed methodology studies would also allow a fuller range of interacting factors that influence HFMC functioning to be explored, including how issues of representation and legitimacy play out in different contexts, and how devolution affects implementation of HSSF in different counties.

There are three potential limitations in our study. First, HSSF preparation activities had begun in some areas before we did our survey (for example the gazetting of HFMCs and a requirement to set up bank accounts for every facility). Although this means that our study was not a totally 'clean’ baseline, it was nevertheless conducted before any HSSF funds were dispersed to health facilities and makes the challenges identified all the more stark in terms of the implications for HSSF impact. Second, it is possible that interviewees gave positive responses to Likert scale questions more often than negative ones in an effort to present themselves in a good light. Third, selection of HFMC community members to be interviewed by the health facility in-charges (authority figures) may have led to a bias in sampling, and more specifically to more positive responses. There were more office holders than non-office holders interviewed, potentially contributing to a relatively well informed set of interviewees. The challenges identified suggest that interviewees were able to raise issues and concerns, but these biases do illustrate the importance of exploring the roles and functioning of HFMCs in more depth using qualitative approaches in future.

## Conclusion

HFMCs have been in place for several decades in Kenya, linked to wider decentralisation and community participation initiatives. Recently there have been moves to expand the role of HFMCs to include management of centrally allocated facility-level funds under the HSSF. In advance of national roll out of HSSF, most HFMCs in Kenya had the basic requirements to operate; that is a functioning committee which met regularly, a bank account (to receive HSSF funds), and motivated HFMC members who had good relationships with the community and the in-charges. However, areas that need more emphasis to ensure that HFMC activities are transparent and members are accountable for how they use the money entrusted to them include training on financial management and targeted supportive supervision. It is also important for members of the wider community to be aware of available funds, activities of the committee, and how they can air their views and contribute to better health services in their communities. The literature suggests that these challenges are likely to be faced by similar committees in other countries.

Once HSSF is fully established, qualitative and quantitative research on how and why HFMCs are adapting to their expanded roles, especially in financial management, would be valuable in informing similar financing mechanisms in Kenya and beyond.

## Endnotes

^a^ An example of each or the three main approaches to asking questions:

• Have you received any benefits for being a committee member? (Options; do not prompt): 1. Allowances (Y/N); 2. Waiver on user fees (Y/N); 3. Priority for treatment (Y/N); 4. Training opportunity (Y/N); 5. Casual employment (Y/N); 6. Goodies (T-shirts, caps, etc.) (Y/N) 7. Opportunity to participate in outreaches/campaigns (Y/N); 8. Other (specify)__________________

• Now I would like to ask you a few questions regarding the roles of the HFMC. Do you think that the following are the roles of the HFMC? (For each of 10 roles, Y/N/Don’t *Know).*

• Note that the 10 roles presented included those that HFMCs were officially expected to have and those that they are expected to have (for example 'Raising the issues they have heard in the community with the facility staff’) and not expected to have (for example 'Employing new government health workers such as nurses’).

• *Now I would like you to think about the statements I am going to read out below. For each one, please tell me whether you strongly agree, agree, are neutral, disagree or strongly disagree (21 statements were read out).*

• *Note that for the 21 statements, some were positively phrased (for example 'I am proud to be a HFMC member’), and others negatively so (for example 'There is too much gossip in this facility about the use of funds’ respectively).*

• *(see*http://resyst.lshtm.ac.uk/resources/nationally-representative-survey-kenya's-public-sectorhealth-centres-and-dispensaries*to view the full questionnaires used).*

## Abbreviations

CHW: Community health worker; CI: Confidence interval; DANIDA: Danish International Development Agency; DHMT: District Health Management Team; DMOH: District Medical Officer of Health; HFMCs: Health Facility Management Committees; HSSF: Health Sector Services Fund; IQR: Inter quartile range; KEMRI: Kenya Medical Research Institute; MOPHS: Ministry of Public Health and Sanitation; MS: Microsoft; SAQ: Self administered questionnaire; USD: United States dollars.

## Competing interests

There are no financial or non-financial competing interests in this manuscript.

## Authors’ contributions

SM and CG designed the study. EW, AO, MT, SM and CG conducted the fieldwork and the analysis. GF and TE provided advice on statistical elements of the study design and analysis. EW wrote the first draft of the paper. All authors commented on several drafts of the paper and approved the final version.

## Authors’ information

EW is an assistant research officer at the KEMRI-Wellcome Trust Programme. Evelyn’s primary responsibilities are to plan and manage data collection, support analysis and write up results from various research projects regarding health systems and governance. Her research interests include health policy planning and financing in developing countries.

AO has worked as a research officer at the KEMRI-Wellcome Trust Research Program in Nairobi. He was in-charge of designing and implementing the HSSF baseline survey in Kenya in 2010 and conducted the pilot survey for direct facility financing in Coast Province in 2007–2008. His main interest is in health-care financing in Kenya and other developing countries.

MT was previously a research officer at KEMRI-Wellcome Trust, conducting analysis on the baseline national survey on the roll out of HSSF. She holds a Master of Science in Health Policy & Management from Harvard School of Public Health.

GF is the Head of Statistics at the KEMRI-Wellcome Trust Research Programme where he has worked since 2003. Prior to that he held positions at the Louisiana Office of Public Health, the UK MRC’s Laboratories in the Gambia, the Demographic and Health Surveys and the London School of Hygiene & Tropical Medicine. Amongst other things he is interested in malaria epidemiology and immunology, large scale public health interventions and the translation of research into policy within health systems.

TE is a Lecturer in Medical Statistics at the London School of Hygiene and Tropical Medicine. Tansy provides design and analytical input to a range of randomised controlled trials and epidemiological studies, mainly for visceral leishmaniasis, trachoma and epilepsy. Teaching responsibilities include tutoring and formal assessment duties for several MSc courses at LSHTM and statistical support for PhD students.

CG is a Senior Lecturer in Health Economics and Policy at the London School of Hygiene and Tropical Medicine, and was based with the KEMRI-Wellcome Trust Research Programme in Nairobi from 2006–2011. Her key research interests include the economics of malaria control, the role of the private sector in health care delivery, and the financing and management of primary health care facilities.

SM is employed by the University of Oxford, and has been based at the KEMRI-Wellcome Trust Research Programme in Kilifi, Kenya, since 1995. Sassy is a senior social scientist at the programme, with her current key research interests including community accountability in health delivery and health research, research ethics, and household access to and use of health facilities.

## Pre-publication history

The pre-publication history for this paper can be accessed here:

http://www.biomedcentral.com/1472-6963/13/404/prepub
